# Supplementary material for: DNA replication licensing factor Cdc6 and Plk4 kinase antagonistically regulate centrosome duplication via Sas-6
Source: Nat Commun. 2017 Apr 27;8:15164. doi: 10.1038/ncomms15164 (PMC5414174; doi:10.1038/ncomms15164)
Supplement: Supplementary Information — Supplementary Figures [file ncomms15164-s1.pdf]

Supplementary Figures

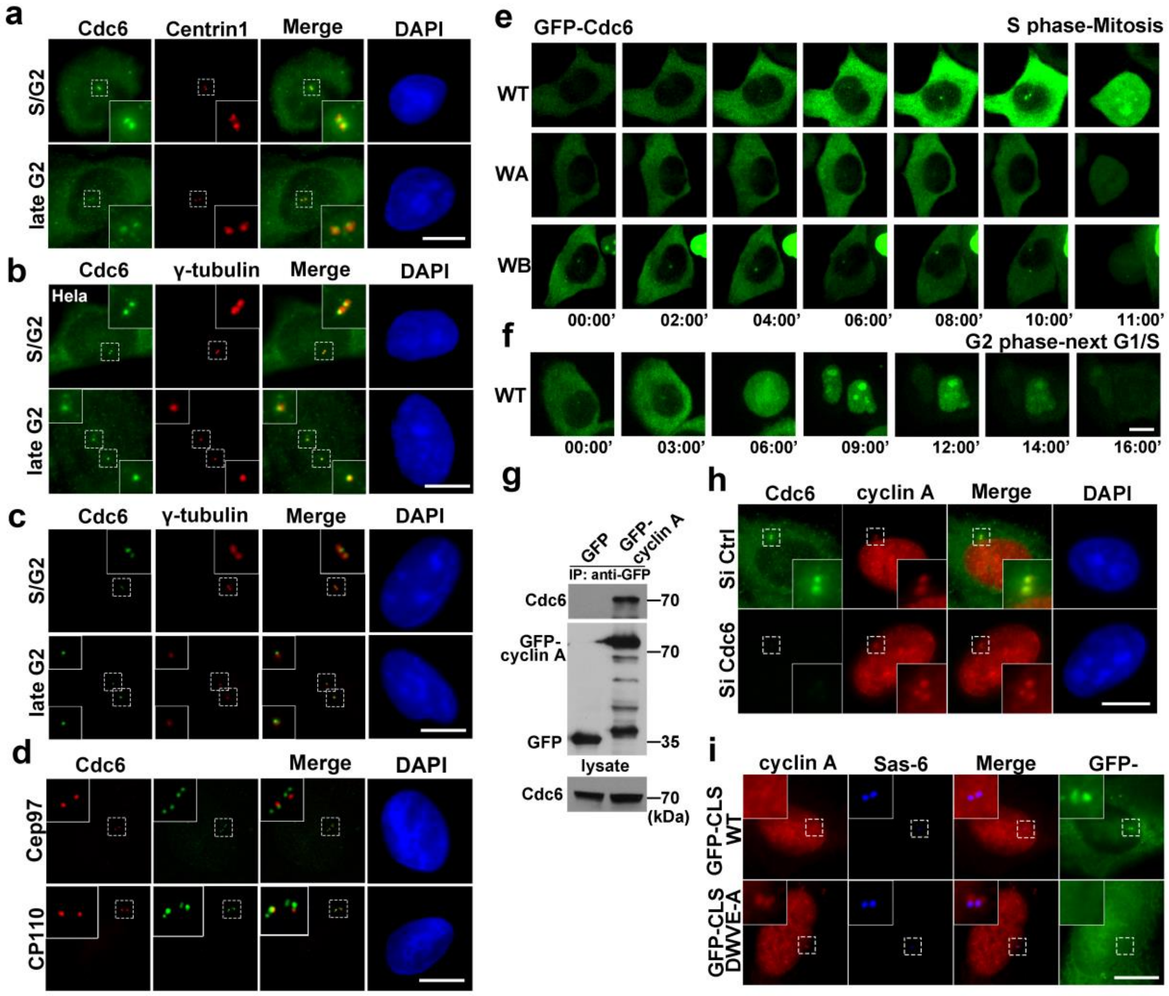

Supplementary Figure 1. Cyclin A mediates the localization of Cdc6 on the proximal side of the centrioles (related to Figure 1). (**a,b,c**) Cdc6 localizes on centrosomes during S and G2 phase. Immunofluorescent labeling of endogenous Cdc6 and centrin1 in U2OS cells (**a**), Cdc6 and γ-tubulin in HeLa cells (**b**) and U2OS cells (**c**). (**d**) Cdc6 localizes to the proximal side of the centriole. Immunofluorescence labeling of endogenous Cdc6 and Cep97 or CP110 in U2OS cells. (**e**) Live-cell imaging of HeLa cells transfected with GFP-tagged Cdc6 WT, WA or WB mutant at the G1/S transition as the starting point (00: 00 represents h: min) to 11 h after G1/S release. (**f**) Live-cell imaging of HeLa cells transfected with GFP-tagged Cdc6 WT at late G2 phase as the starting point (00: 00 represents h: min) to 16 h (until next G1/S) to show the cell cycle dependent centrosome localization of Cdc6. (**g**) Cdc6 interacts with cyclin A in cells. HEK293 cells were transfected with GFP-cyclin A, and

the total cell extract was used for immunoprecipitation with a GFP antibody and probed with Cdc6 and GFP antibodies. (**h**) Centrosome localization of cyclin A was not affected by Cdc6 depletion. Immunofluorescence of Cdc6 and cyclin A in U2OS cells transfected with control or Cdc6 siRNA. (**i**) Overexpression of the CLS fragment replaced endogenous cyclin A at the centrosome. U2OS cells expressing GFP-tagged CLS or CLS mutant DWVE-A were stained with Cdc6 and Sas-6 antibodies. DNA was stained with DAPI. Scale bars, 10 μm. Insets in **a**-**d, h** and **i** are high-magnification views of the regions indicated in the low-magnification images.

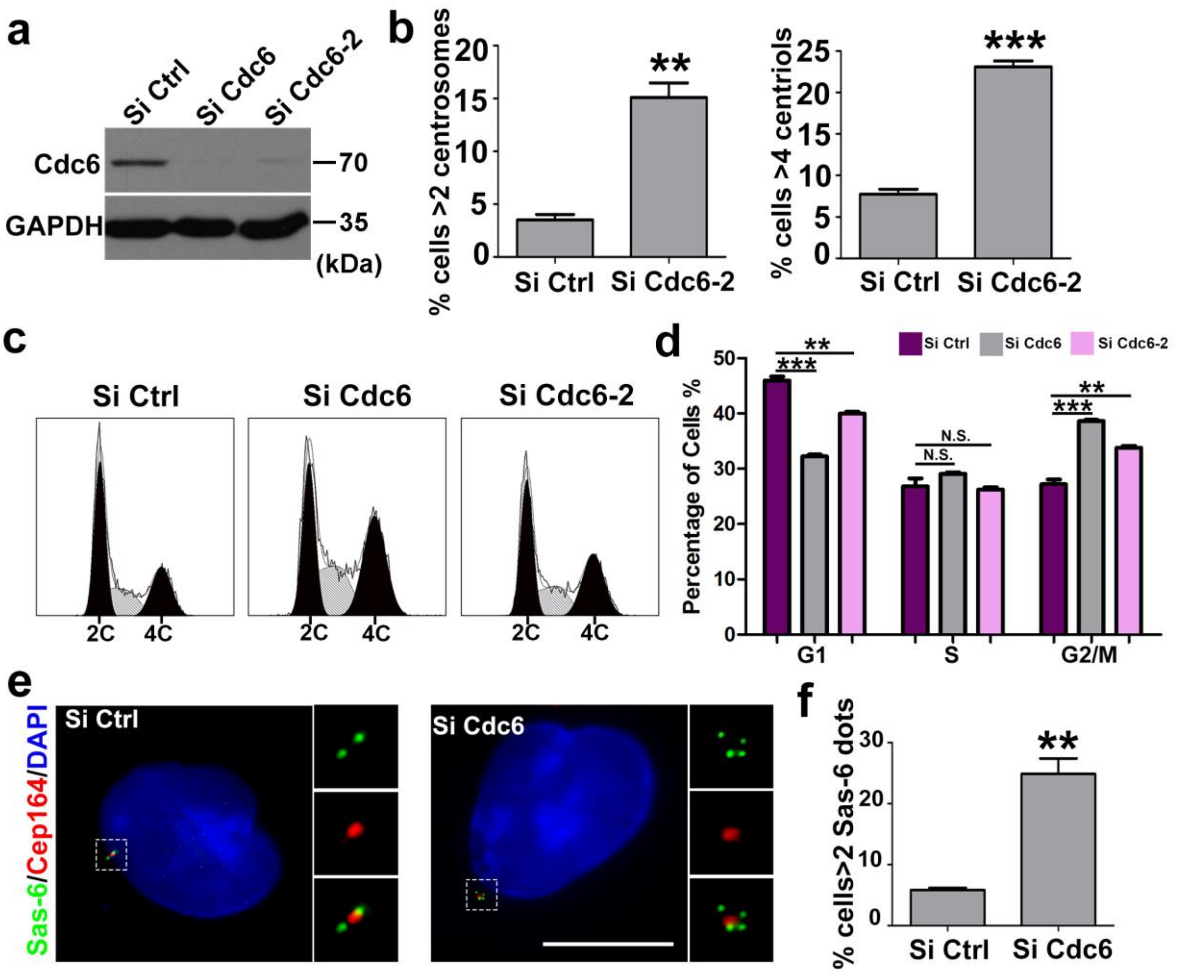

Supplementary Figure 2. Cdc6 depletion results in centriole amplification (related to Figure 2). (**a**) Depletion efficiency of two different Cdc6 siRNAs in U2OS cells was shown by western blotting with Cdc6 and GAPDH antibodies. (**b**) Cdc6 depletion induces centriole and centrosome amplification. Quantitation of cells with >2 centrosomes or >4 centrioles in Cdc6-2 siRNA transfected cells. Approximately 300 cells were counted per sample, and three independent experiments were conducted. (**c**) Cdc6 depletion results in G2/M arrest. Flow cytometry analysis of the cell cycle in Cdc6-depleted cells. (**d**) Quantitation of cells in G1, S and G2/M phase in **c** from three independent experiments. (**e**) Cdc6 depletion induces multiple procentrioles formation. Immunofluorescence of Sas-6 (green) and Cep164 (red) in U2OS cells transfected with control or Cdc6 siRNA. (**f**) Quantitation of cells with >2 Sas-6-positive dots in **e**. Approximately 300 cells were counted per sample, and three independent experiments were conducted. The statistical data in **b,d** and **f** are presented as means ± SD. $^{**}P < 0.01$ and $^{***}P < 0.001$; N.S., no significant difference (Student's *t*-test). DNA was stained with DAPI. Scale bars, 10 μm. Insets in **e** are high-magnification views of the regions indicated in the low-magnification images.

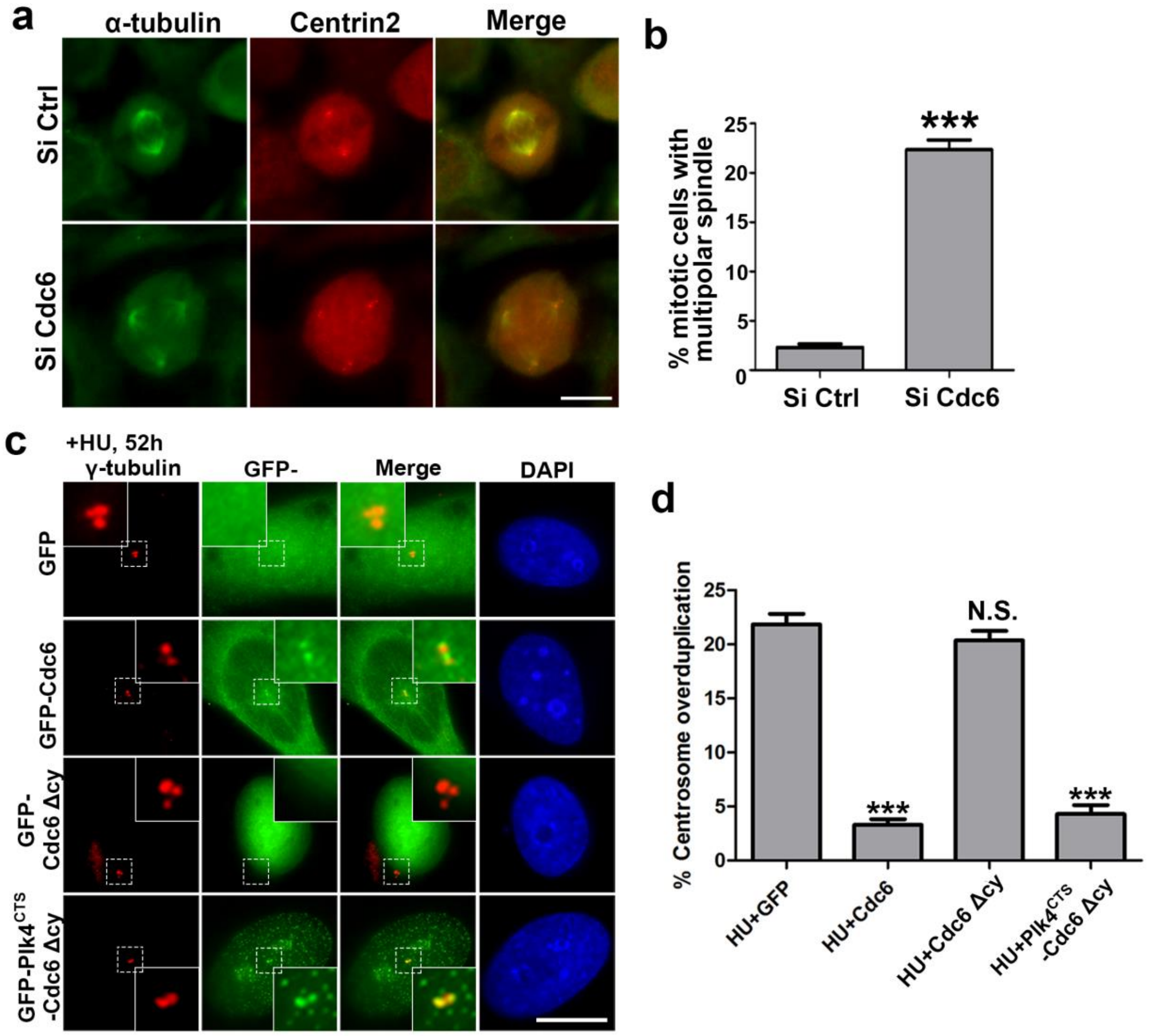

Supplementary Figure 3. Cdc6 depletion results in multipolar spindle formation, and centrosomal Cdc6 inhibits centrosome over-duplication (related to Figure 2). (**a**) Cdc6 depletion induces multipolar spindle formation during mitosis. Immunofluorescence of α-tubulin and centrin2 in Hela cells transfected with control or Cdc6 siRNA. (**b**) Quantitation of mitotic cells with multipolar spindles. Approximately 300 cells were counted per sample, and three independent experiments were conducted. (**c**) Centrosomal localization of Cdc6 is required for the inhibition of HU-induced centrosome over-duplication. U2OS cells transfected with GFP, GFP-tagged Cdc6, GFP-tagged Cdc6 Δcy or GFP-tagged Plk4^{CTS}-Cdc6 Δcy were treated with 16 mM HU for 52 h to allow centriole amplification. The cells were then stained with a γ-tubulin antibody. (**d**) Quantitation of cells with >2 centrosomes in **c**. Approximately 300 cells were counted per sample, and three independent experiments were conducted. The statistical data in **b** and **d** are presented as means ± SD.

***$P < 0.001$ (Student's *t*-test). DNA was stained with DAPI. Scale bars, 10 μm. Insets in **c** are high-magnification views of the regions indicated in the low-magnification images.

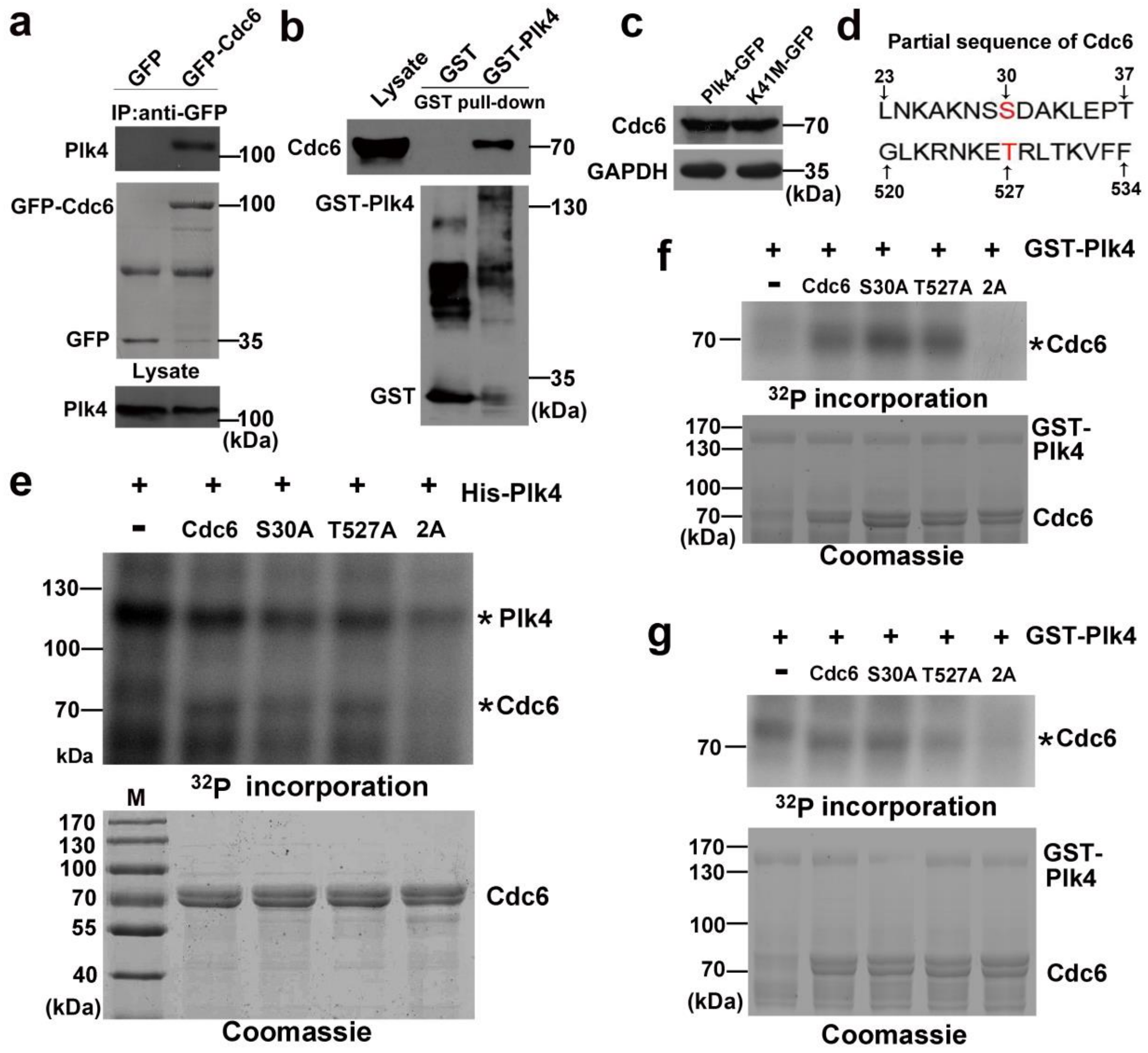

Supplementary Figure 4. Plk4 binds and phosphorylates Cdc6 (related to Figure 4). (**a**) GFP tagged Cdc6 interacts with Plk4 in cells. HEK293 cells were transfected with GFP-tagged Cdc6, and the total cell extract was immunoprecipitated with a GFP antibody and probed with Plk4 and GFP antibodies. (**b**) Cdc6 interacts with Plk4 in a Plk4 pull-down assay. Sepharose beads coupled with bacterially expressed GST or GST-tagged Plk4 were incubated with HEK293 cell lysates, isolated and analyzed by western blotting using Cdc6 and GST antibodies. (**c**) GFP-tagged Plk4 K41M mutant overexpression does not affect the Cdc6 protein level. HEK293 cells transfected with GFP-tagged Plk4 or GFP-tagged Plk4 K41M were lysed and probed with Cdc6 and GAPDH antibodies. (**d**) Sequence analysis indicated that serine 30 and threonine 527 (indicated in red) in Cdc6 are the potential Plk4

phosphorylation sites. (**e**) Plk4 phosphorylates Cdc6 on serine 30 and threonine 527 in vitro. Purified His-tagged Plk4 from the baculovirus/insect cell expression system was incubated with His-tagged Cdc6, Cdc6 S30A, T527A or double mutation 2A (S30A and T527A) in the presence of [γ-^{32}P]-ATP, followed by autoradiography. His-Plk4 alone without incubation with Cdc6 served as the control in the in vitro kinase assay. Coomassie blue staining shows the loadings of His-tagged Cdc6, S30A, T527A and 2A proteins. Asterisk indicates phosphorylated Cdc6 or Plk4. Molecular weight markers are indicated on the left of the image. (**f,g**) Two independent experiments of the in vitro kinase assay by GST-tagged Plk4. Purified GST-tagged Plk4 protein was incubated with purified His-tagged Cdc6, Cdc6 S30A, Cdc6 T527A or double mutation 2A proteins in the presence of [γ-^{32}P]-ATP, followed by autoradiography. Coomassie blue staining showed the loadings of GST-tagged Plk4 and His-tagged Cdc6 WT and Cdc6 mutants proteins. Asterisk indicates phosphorylated Cdc6. Molecular weight markers are indicated on the left of the image.

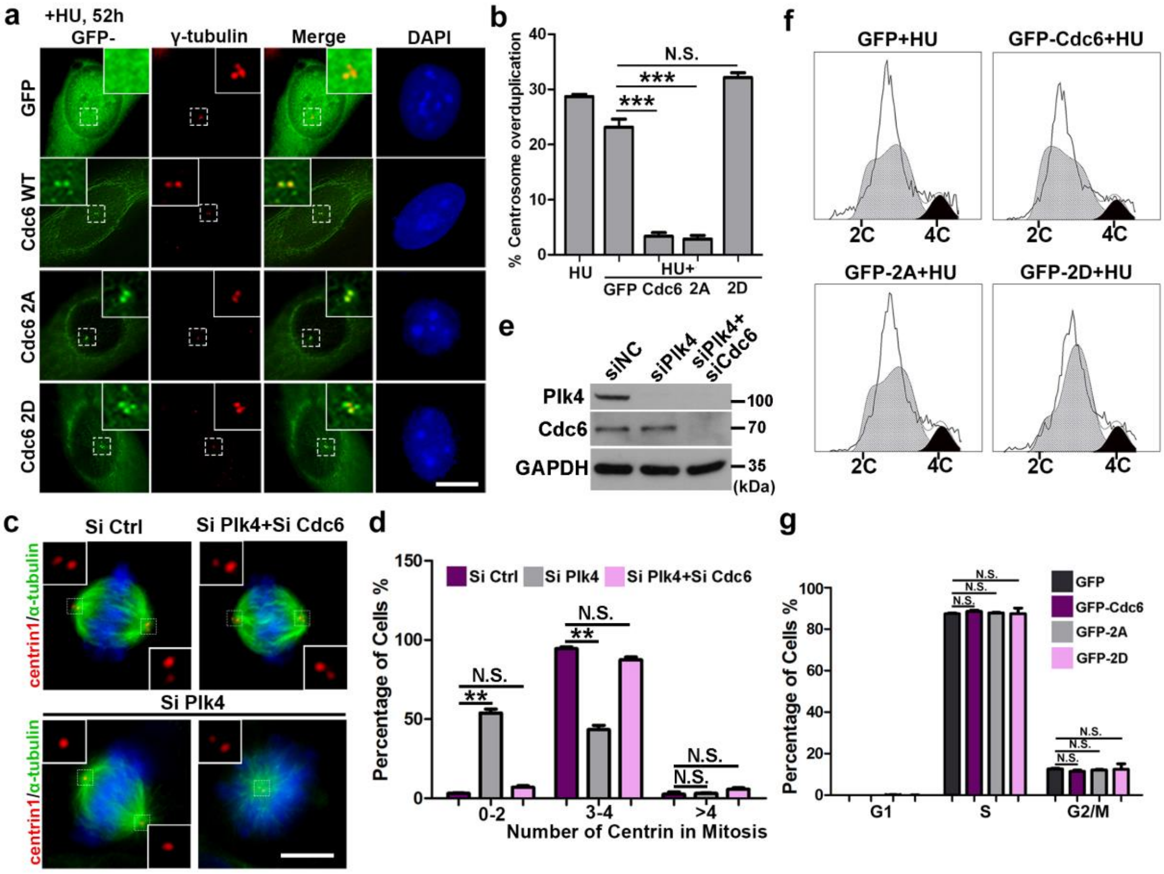

Supplementary Figure 5. Cdc6 inhibits centrosome over-duplication downstream of Plk4 (related to Figure 5). (**a**) Cdc6 WT or 2A mutant, but not 2D mutant, inhibits HU-induced centrosome over-duplication. U2OS cells were transfected with GFP, GFP-tagged Cdc6 WT, GFP-tagged Cdc6 2A or 2D mutant, and treated with 16 mM HU for 52 h to allow centriole amplification. The cells were then stained with a γ-tubulin antibody. (**b**) Quantitation of cells with >2 centrosomes in **a**. Approximately 300 cells were counted per sample, and three independent experiments were conducted. (**c**) Simultaneous depletion of Cdc6 and Plk4 restored the mitotic centriole number which was decreased after Plk4 depletion. U2OS cells were transfected with control or Plk4 siRNA; or co-transfected with Cdc6 siRNA and Plk4 siRNA. The cells were then stained with α-tubulin and centrin1 antibodies. (**d**) Quantitation of centrin1-positive dots number in mitotic cells in **c**. Approximately 100 cells were counted per sample, and three independent experiments were conducted. (**e**) U2OS cells were depleted of Plk4 or simultaneously depleted of Plk4 and Cdc6 by siRNAs, and the knockdown efficiency was determined by western blotting with

Plk4, Cdc6 and GAPDH antibodies. (**f**) HU-induced S phase arrest was not influenced by Cdc6 WT, 2A, or 2D mutant overexpression. Flow cytometry analysis of U2OS cells transfected with GFP, GFP-tagged Cdc6 WT, GFP-tagged Cdc6 2A or 2D mutant, and treated with 16 mM HU for 52 h to allow centriole amplification. (**g**) Quantitation of cells in G1, S and G2/M phase from three independent experiments. The statistical data in **b,d** and **g** are presented as means ± SD. $^{**}P < 0.01$ and $^{***}P < 0.001$; N.S., no significant difference (Student's *t*-test). DNA was stained with DAPI. Scale bars, 10 μm. Insets in **a** and **c** are high-magnification views of the regions indicated in the low-magnification images.

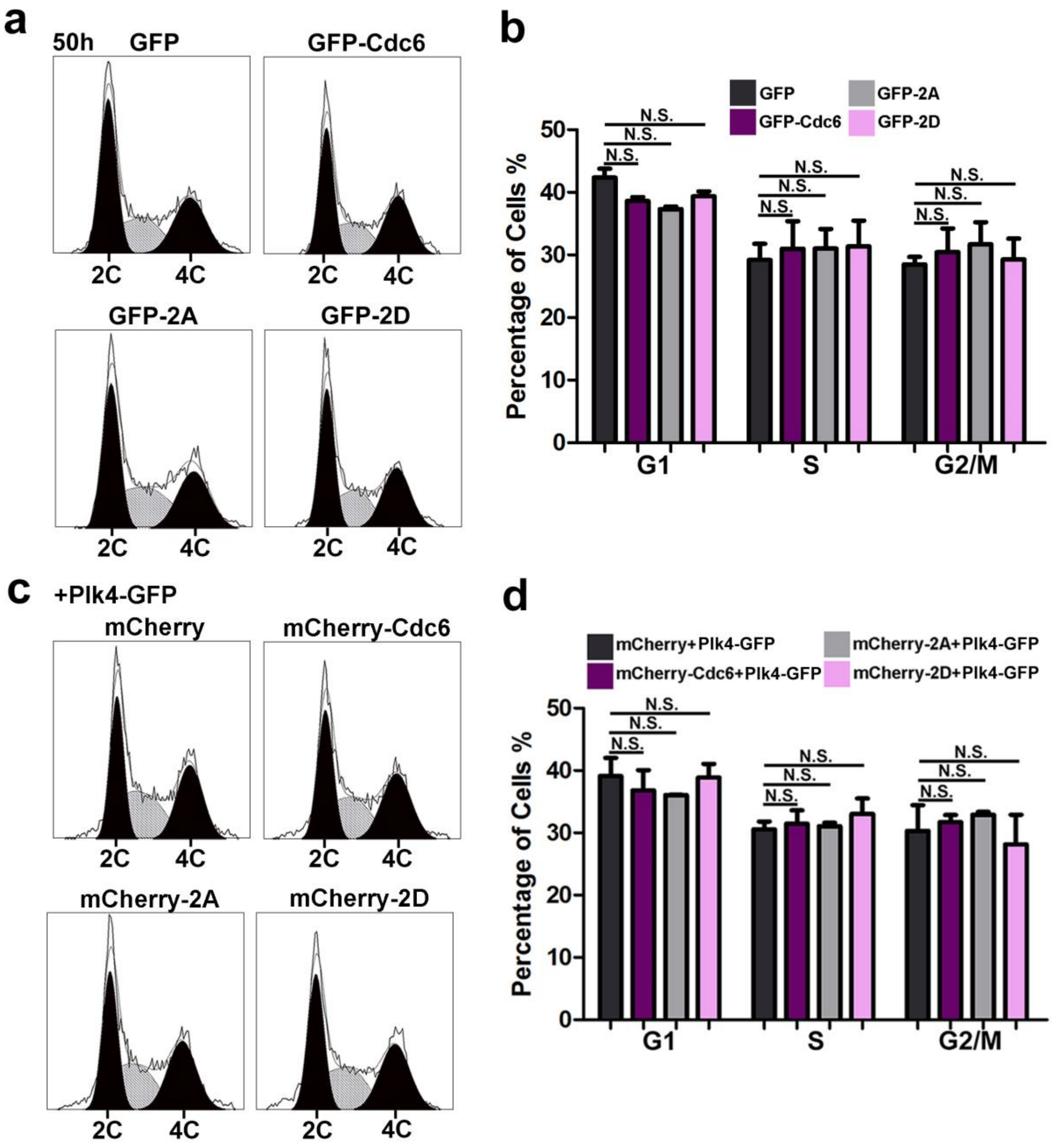

Supplementary Figure 6. Cdc6 or Plk4 overexpression does not affect the cell cycle (related to Figure 5). (**a**) GFP tagged Cdc6 WT, 2A or 2D mutant overexpression does not affect the cell cycle. Flow cytometry analysis of U2OS cells transfected with GFP, GFP-tagged Cdc6 WT, and GFP-tagged Cdc6 2A or 2D mutant for 50 h. (**b**) Quantitation of cells in G1, S and G2/M phase from three independent experiments. (**c**) Co-overexpression of mCherry-tagged Cdc6 WT, 2A or 2D mutant with GFP-tagged Plk4 does not affect the

cell cycle. Flow cytometry analysis of U2OS cells transfected with GFP-tagged Plk4 and mCherry-tagged Cdc6 WT, 2A or 2D mutant for 40 h. (**d**) Quantitation of cells in G1, S and G2/M phase from three independent experiments. The statistical data in **b** and **d** are presented as means ± SD. N.S., no significant difference (Student's *t*-test).

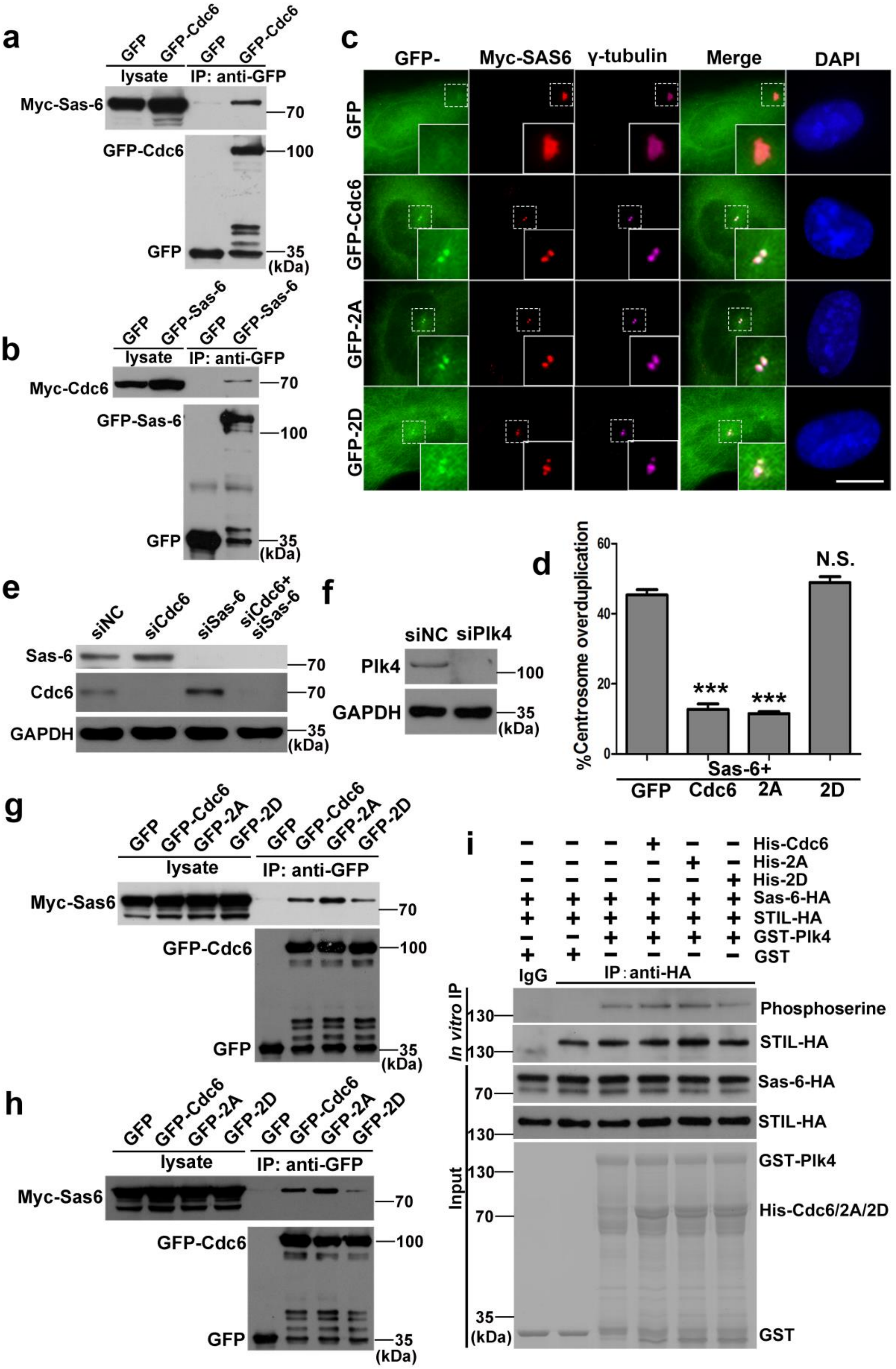
a
GFP
GFP-Cdc6
GFP
GFP-Cdc6
lysate
IP: anti-GFP
Myc-Sas-6
70
GFP-Cdc6
100
GFP
35
(kDa)
b
GFP
GFP-Sas-6
GFP
GFP-Sas-6
lysate
IP: anti-GFP
Myc-Cdc6
70
GFP-Sas-6
100
GFP
35
(kDa)
c
GFP-
Myc-SAS6
γ-tubulin
Merge
DAPI
GFP
GFP-Cdc6
GFP-2A
GFP-2D
d
%Centrosome overduplication
60
40
20
0
N.S.
***
***
Sas-6+
GFP
Cdc6
2A
2D
e
siNC
siCdc6
siSas-6
siCdc6+ siSas-6
Sas-6
Cdc6
GAPDH
70
70
35
(kDa)
f
siNC
siPlk4
Plk4
100
GAPDH
35
(kDa)
g
GFP
GFP-Cdc6
GFP-2A
GFP-2D
GFP
GFP-Cdc6
GFP-2A
GFP-2D
lysate
IP: anti-GFP
Myc-Sas6
70
GFP-Cdc6
100
GFP
35
(kDa)
h
GFP
GFP-Cdc6
GFP-2A
GFP-2D
GFP
GFP-Cdc6
GFP-2A
GFP-2D
lysate
IP: anti-GFP
Myc-Sas6
70
GFP-Cdc6
100
GFP
35
(kDa)
i
His-Cdc6
His-2A
His-2D
Sas-6-HA
STIL-HA
GST-Plk4
GST
IgG
IP : anti-HA
In vitro IP
Input
Phosphoserine
STIL-HA
Sas-6-HA
STIL-HA
GST-Plk4
His-Cdc6/2A/2D
GST
130
130
70
130
130
70
35
(kDa)

Supplementary Figure 7. Plk4 promotes centriole amplification through releasing Sas-6 from Cdc6 inhibition (related to Figure 6 and Figure 7). (**a**) GFP-tagged Cdc6 interacts with Myc-tagged Sas-6 in cells. HEK293 cells were co-transfected with GFP-Cdc6 and Myc-Sas-6. The whole cell extract was immunoprecipitated with GFP antibody probed with Myc and GFP antibodies. (**b**) GFP-tagged Sas-6 interacts with Myc-tagged Cdc6 in cells. HEK293 cells were co-transfected with GFP-tagged Sas-6 and Myc-tagged Cdc6. The whole cell extract was immunoprecipitated with GFP antibody, and probed with Myc and GFP antibodies. (**c**) Cdc6 WT or Cdc6 2A mutant, but not Cdc6 2D mutant, suppresses the Sas-6-induced centriole amplification. Myc-tagged Sas-6 was co-transfected with GFP, GFP-tagged Cdc6 WT, GFP-tagged Cdc6 2A or 2D mutant for 28 h. The cells were then stained with Myc and γ-tubulin antibodies. (**d**) Quantitation of cells with centrosome over-duplication in **d**. Approximately 300 cells were counted per sample, and three independent experiments were conducted. (**e**) U2OS cells were individually or simultaneously depleted of Cdc6 and/or Sas-6 by siRNAs, and the knockdown efficiency was determined by western blotting with Sas-6, Cdc6 and GAPDH antibodies. (**f**) Depletion efficiency of Plk4 siRNA in U2OS cells is shown by western blotting with Plk4 and GAPDH antibodies. (**g,h**) Two independent experiments of Cdc6 phosphorylation inhibiting the interaction between Cdc6 and Sas-6 in cells. HEK293 cells co-transfected with Myc-Sas-6 and GFP-tagged Cdc6, 2A or 2D mutant were immunoprecipitated with GFP antibody, and probed by Myc and GFP antibodies. (i) Phosphorylation status of STIL in the in vitro protein-protein binding assay of Sas-6 and STIL. HA-Sas-6 protein was incubated with indicated purified proteins in the presence of HA-STIL protein in kinase reaction buffer. HA-STIL was then immunoprecipitated by HA antibody, and the bound proteins were analyzed with STIL and Phosphoserine antibodies. The loadings of indicated proteins are shown by Coomassie blue staining. The statistical data in **d** is presented as means ± SD. ****P* < 0.001; N.S., no significant difference (Student's *t*-test). DNA was stained with DAPI. Scale bars, 10 μm. Insets in **c** are high-magnification views of the regions indicated in the low-magnification images.

Uncropped western blots and gels

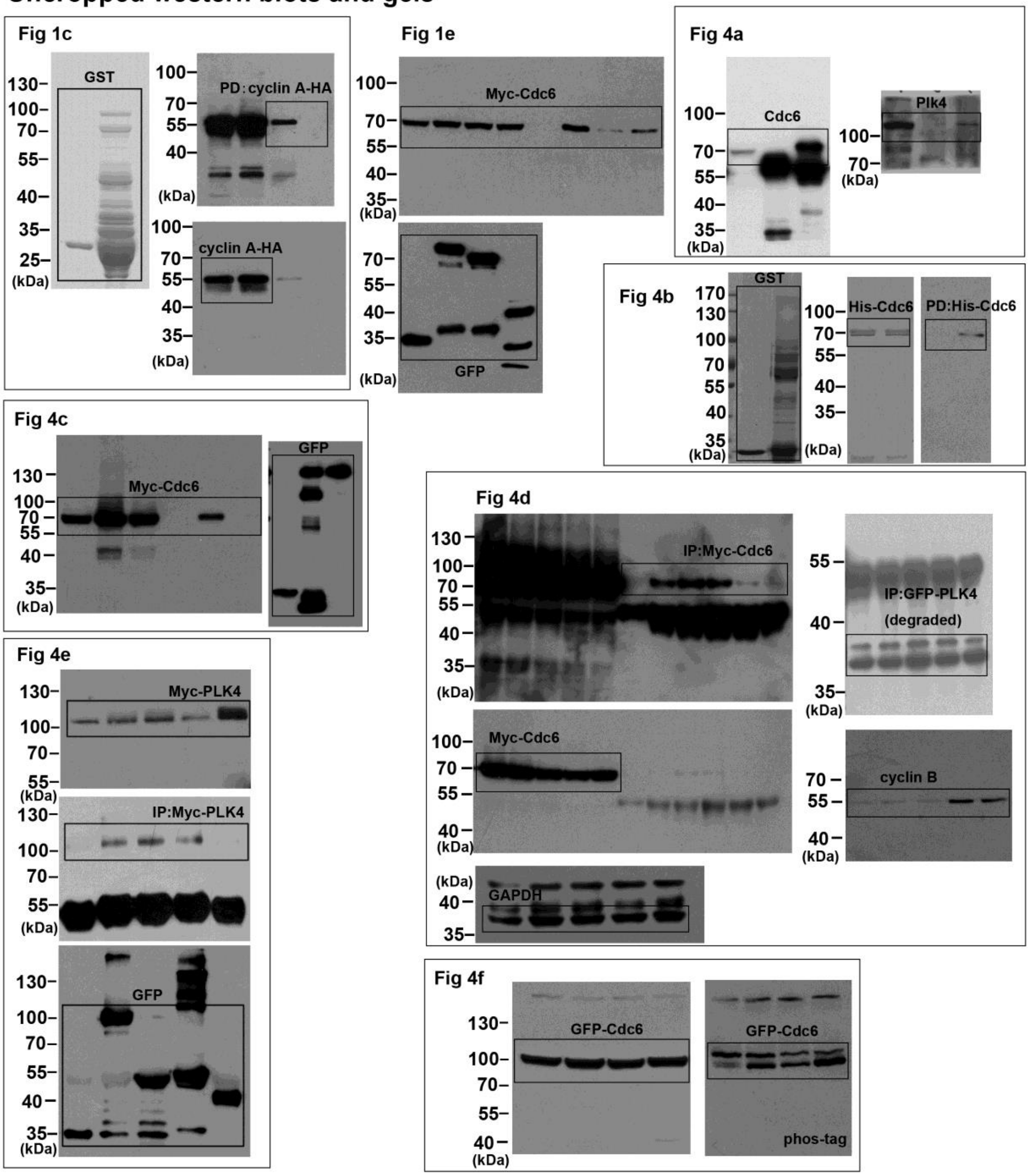

Supplementary Figure 8. Uncropped scans of blots and gels from Fig. 1c, Fig. 1e and Fig. 4a-f.

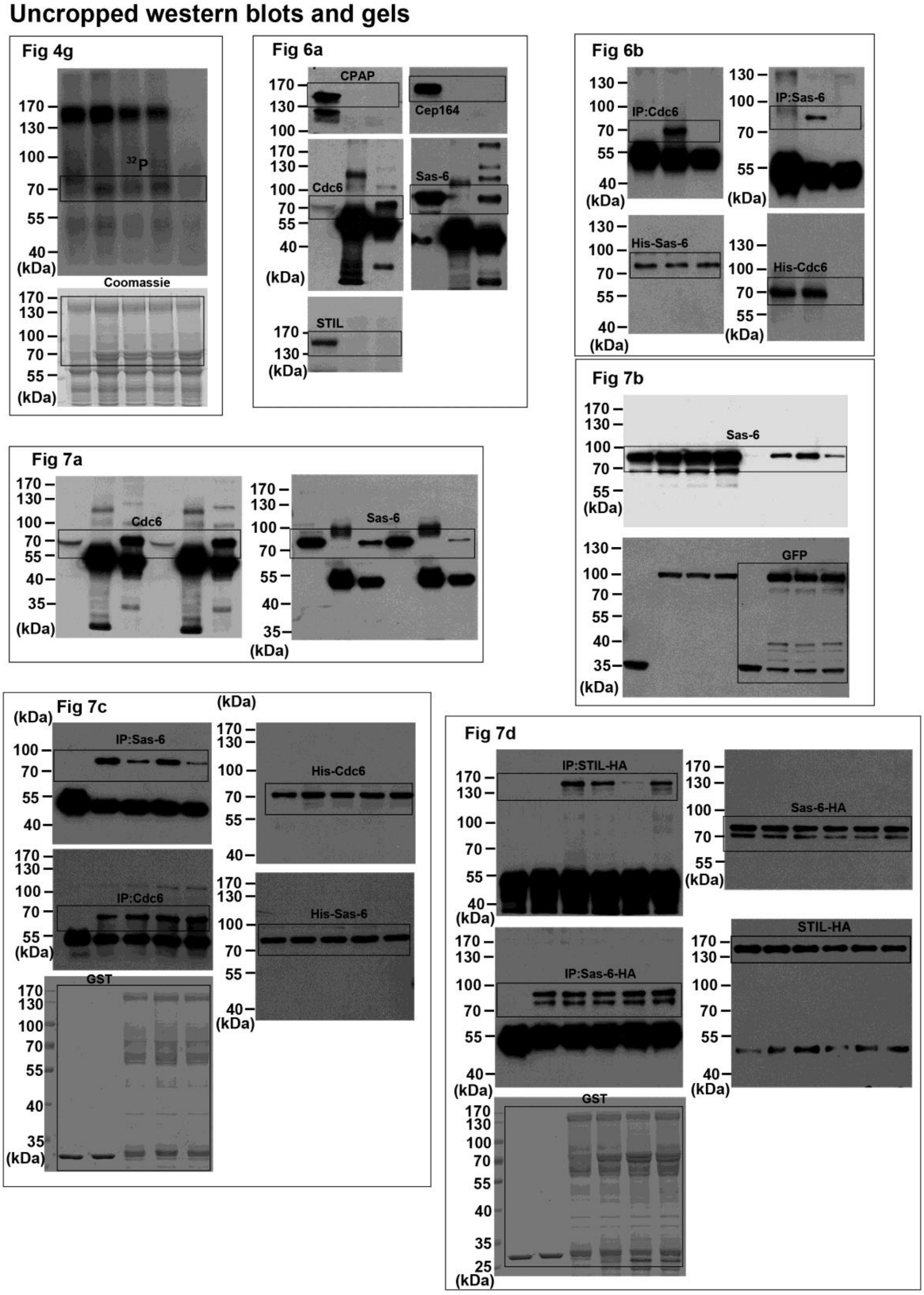

Supplementary Figure 9. Uncropped scans of blots and gels from Fig. 4g, Fig. 6a, Fig. 6b and Fig. 7a-d.

Uncropped western blots and gels

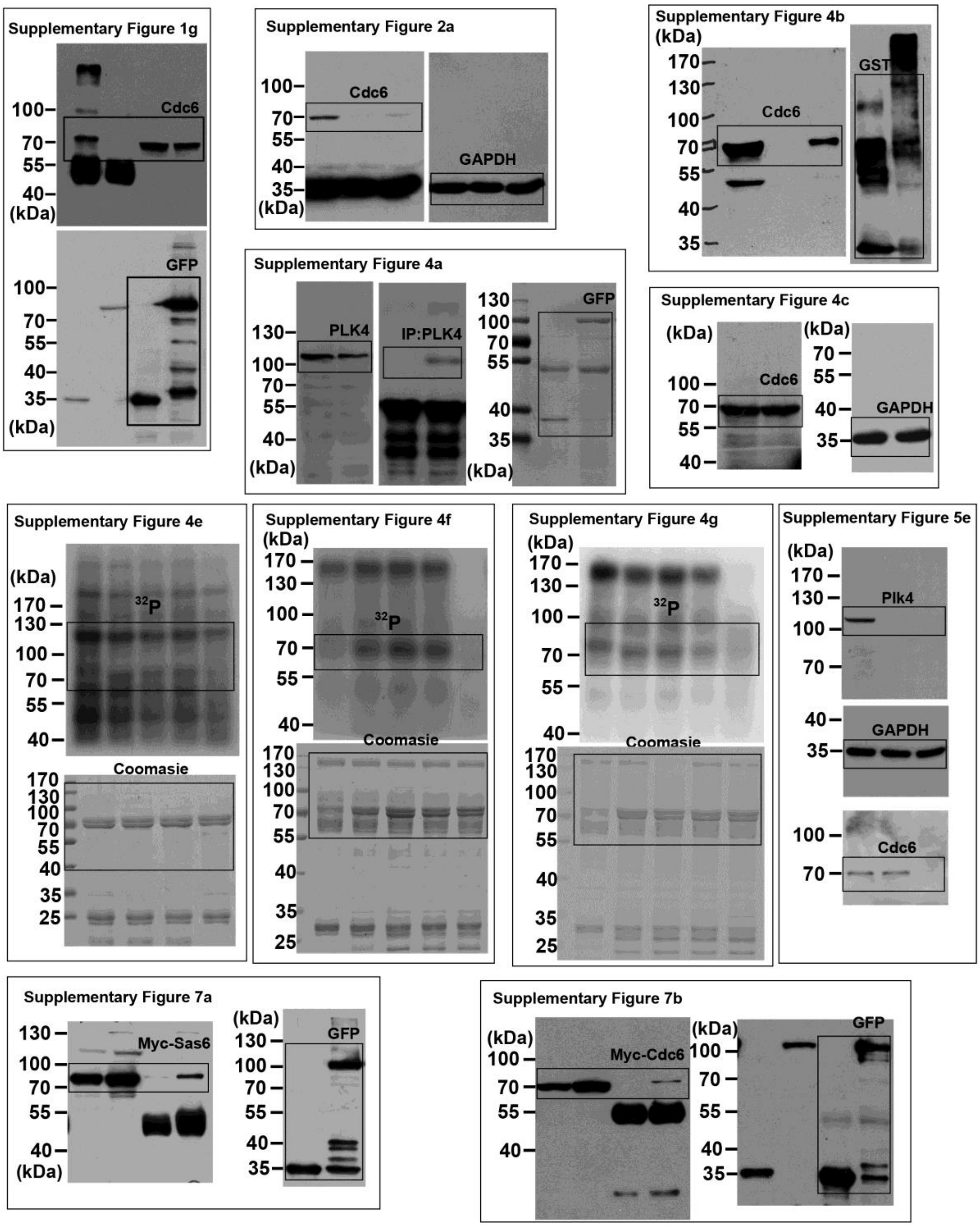

Supplementary Figure 10. Uncropped scans of blots and gels from Supplementary Fig. 1g, Supplementary Fig. 2a, Supplementary Fig. 4a-c, Supplementary Fig. 4e-g, Supplementary Fig. 5e and Supplementary Fig. 7a,b.

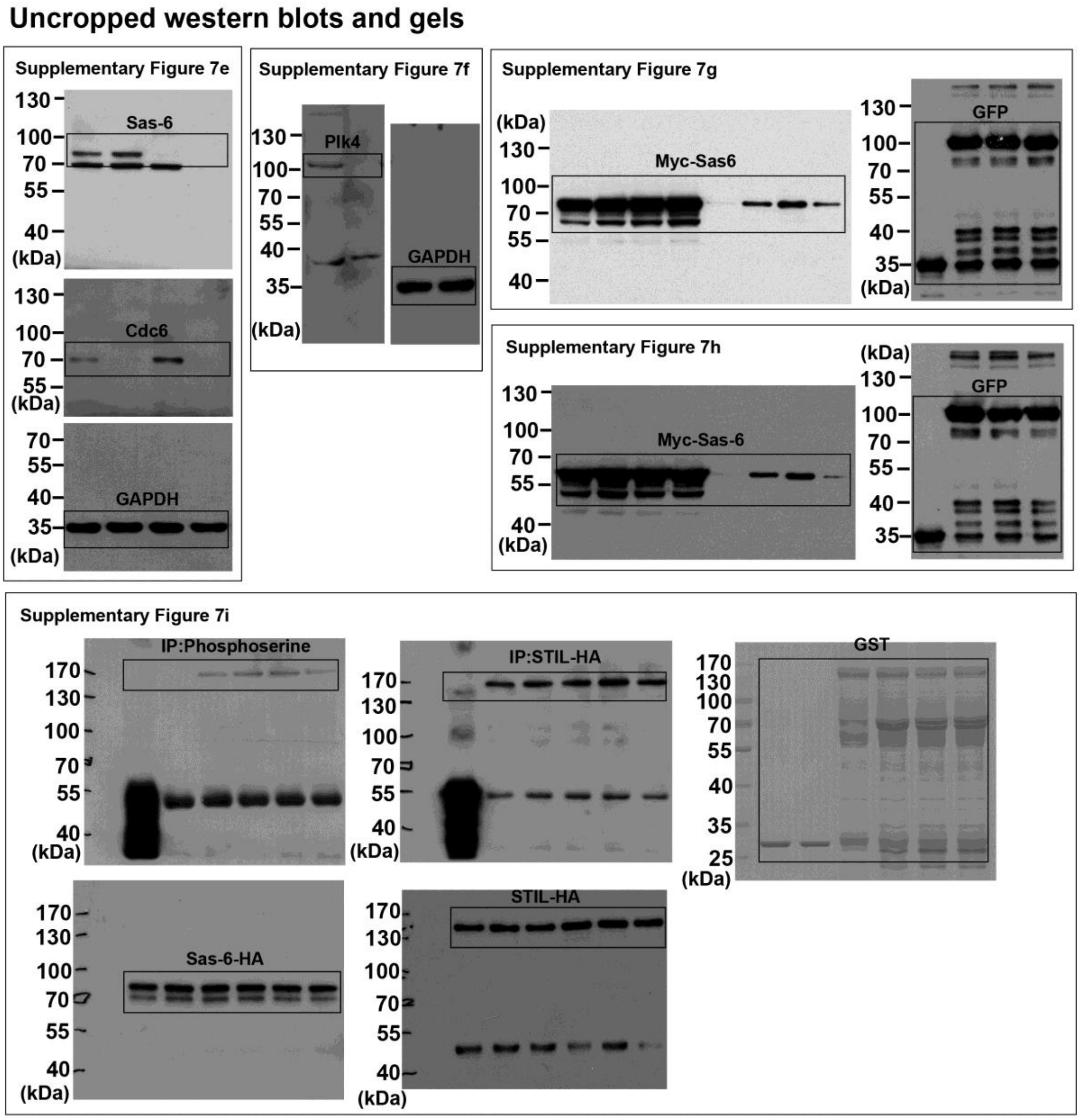

Supplementary Figure 11. Uncropped scans of blots and gels from Supplementary Fig. 7e-i.
